# The impact of damaging epilepsy and cardiac genetic variant burden in sudden death in the young

**DOI:** 10.1186/s13073-024-01284-w

**Published:** 2024-01-16

**Authors:** Megan J. Puckelwartz, Lorenzo L. Pesce, Edgar J. Hernandez, Gregory Webster, Lisa M. Dellefave-Castillo, Mark W. Russell, Sarah S. Geisler, Samuel D. Kearns, Felix Karthik, Susan P. Etheridge, Tanner O. Monroe, Tess D. Pottinger, Prince J. Kannankeril, M. Benjamin Shoemaker, Darlene Fountain, Dan M. Roden, Meghan Faulkner, Heather M. MacLeod, Kristin M. Burns, Mark Yandell, Martin Tristani-Firouzi, Alfred L. George, Elizabeth M. McNally

**Affiliations:** 1 Department of Pharmacology, Feinberg School of Medicine, Northwestern University, Chicago, IL USA; 2 Center for Genetic Medicine, Feinberg School of Medicine, Northwestern University, Chicago, IL USA; 3 Biomedical Informatics, University of Utah, Salt Lake City, UT USA; 4 Division of Cardiology, Department of Pediatrics, Ann & Robert H. Lurie Children’s Hospital of Chicago, Chicago, IL USA; 5 Department of Pediatrics, University of Michigan, Ann Arbor, MI USA; 6 Division of Pediatric Cardiology, University of Utah, Salt Lake City, UT USA; 7 Department of Pediatrics, Vanderbilt University Medical Center, Nashville, TN USA; 8 Department of Medicine, Division of Cardiovascular Medicine, Vanderbilt University Medical Center, Nashville, TN USA; 9 Departments of Medicine, Pharmacology, and Biomedical Informatics, Vanderbilt University Medical Center, Nashville, TN USA; 10 Michigan Public Health Institute, Okemos, MI USA; 11 Data Coordinating Center, SDY Case Registry, Okemos, MI USA; 12 Division of Cardiovascular Sciences, National Heart, Lung, and Blood Institute, National Institutes of Health, Bethesda, MD USA; 13 Department of Human Genetics, University of Utah, Salt Lake City, UT USA

**Keywords:** Sudden death in the young, Genome sequencing, Epilepsy, Arrhythmia, Cardiomyopathy, Gene burden

## Abstract

**Background:**

Sudden unexpected death in children is a tragic event. Understanding the genetics of sudden death in the young (SDY) enables family counseling and cascade screening. The objective of this study was to characterize genetic variation in an SDY cohort using whole genome sequencing.

**Methods:**

The SDY Case Registry is a National Institutes of Health/Centers for Disease Control and Prevention surveillance effort to discern the prevalence, causes, and risk factors for SDY. The SDY Case Registry prospectively collected clinical data and DNA biospecimens from SDY cases < 20 years of age. SDY cases were collected from medical examiner and coroner offices spanning 13 US jurisdictions from 2015 to 2019. The cohort included 211 children (median age 0.33 year; range 0–20 years), determined to have died suddenly and unexpectedly and from whom DNA biospecimens for DNA extractions and next-of-kin consent were ascertained. A control cohort consisted of 211 randomly sampled, sex- and ancestry-matched individuals from the 1000 Genomes Project. Genetic variation was evaluated in epilepsy, cardiomyopathy, and arrhythmia genes in the SDY and control cohorts. American College of Medical Genetics/Genomics guidelines were used to classify variants as pathogenic or likely pathogenic. Additionally, pathogenic and likely pathogenic genetic variation was identified using a Bayesian-based artificial intelligence (AI) tool.

**Results:**

The SDY cohort was 43% European, 29% African, 3% Asian, 16% Hispanic, and 9% with mixed ancestries and 39% female. Six percent of the cohort was found to harbor a pathogenic or likely pathogenic genetic variant in an epilepsy, cardiomyopathy, or arrhythmia gene. The genomes of SDY cases, but not controls, were enriched for rare, potentially damaging variants in epilepsy, cardiomyopathy, and arrhythmia-related genes. A greater number of rare epilepsy genetic variants correlated with younger age at death.

**Conclusions:**

While damaging cardiomyopathy and arrhythmia genes are recognized contributors to SDY, we also observed an enrichment in epilepsy-related genes in the SDY cohort and a correlation between rare epilepsy variation and younger age at death. These findings emphasize the importance of considering epilepsy genes when evaluating SDY.

**Supplementary Information:**

The online version contains supplementary material available at 10.1186/s13073-024-01284-w.

## Background

Sudden death in children has immediate and sustained medical and emotional consequences for affected families. Elucidating the etiology of sudden death can inform risk for surviving family members. The Sudden Death in the Young (SDY) Case Registry was created as a joint initiative between the National Institutes of Health (NIH) and the Centers for Disease Control and Prevention (CDC) to enable a population-based surveillance of SDY and facilitate research through the collection of clinical data and biospecimens [1]. The SDY Case Registry engaged public health agencies in thirteen diverse US sites to collect information on children, ages 0–20 years, who died suddenly and unexpectedly. Data from the SDY Case Registry indicates that sudden death mortality rates are greater for infants less than 1 year (120/100,000 live births) than for children 1–17 years (1.9/100,000 children) [2]. SDY can be attributed to primary cardiac causes including arrhythmias, cardiomyopathies, congenital heart defects, and vascular disease as well as noncardiac causes such as epilepsy. While extensive work has established social and environmental factors that contribute to sudden infant death syndrome (SIDS), the genetic contributors to sudden unexpected infant death (SUID), sudden cardiac death, and sudden unexpected death in epilepsy (SUDEP) remain incompletely described.

There is increasing evidence that SDY has a genetic component and postmortem genetic screening can aid in identifying the cause of death. Testing of genes implicated in cardiac rhythm and function identifies a pathogenic or likely pathogenic (P/LP) variant in 10–25% of individuals with sudden unexplained death < 40 years old [3, 4]. Approximately 4% of SIDS may be due to clinically actionable genetic cardiac causes [5]. In a cohort of sudden unexpected death in pediatrics (SUPD), contributory genetic variants were identified in 11% of decedents, and the authors also found an excess burden of rare, damaging SUDEP gene variants compared to a control group [6]. We previously used genome sequencing (GS) to analyze an SDY cohort (*n* = 103) covering a larger age range (> 1 and < 44 years) and found ~13% carried a pathogenic/likely pathogenic (P/LP) variant in an arrhythmia or cardiomyopathy gene. In that cohort, younger decedents carried an excess of suspicious variants of uncertain significance (VUS) and P/LP variants compared to a control population. Furthermore, for decedents > 2 years of age at death, a younger age associated with harboring a greater number of rare cardiac variants [7]. Here, we interrogated the genomes of 211 decedents (≤ 20 years old) ascertained through the NIH/CDC SDY Case Registry with a median age at death of 0.33 year, with an analysis of genes associated with epilepsy, cardiomyopathies, and arrhythmias.

## Methods

### SDY Case Registry

The SDY Case Registry is a collaboration between the NIH, CDC and the Michigan Public Health Institute [1]. The SDY Case Registry cases included in this study were aggregated during 2015–2019 from 13 participating states and jurisdictions in cooperation with local public health agencies, including medical examiner and coroner offices. The SDY Case Registry includes children from birth to 20 years. Inclusion requires that death be sudden, within 24 h of first symptom or death in a hospital setting after resuscitation from a cardiac arrest, and unexpected which includes subjects in good health or with an illness not reasonably expected to cause death [8]. Exclusion criteria are accidental death with only and obvious cause, homicide, suicide, overdose, and terminal illness (Additional File 1: Figure S1). From 2015 to 2019, 3598 cases met the inclusion criteria of a sudden and unexpected death and were eligible for next of kin consent. In total, 230 decedents from the SDY Case Registry were enrolled in this genetic study and underwent genome sequencing (6%). Of the 230 initially identified, 19 were excluded: 4 due to failed inclusion criteria and 15 due to failed sequencing where no additional sample was available [9]. Clinical data were collected using the National Fatality Review Case Reporting System (NFR-CRS). The SDY Case Registry also received information from local sources, including the death scene report including the Sudden Unexpected Infant Death Investigation Reporting Form (SUIDIRF) if the child was under the age of 1, the autopsy, birth certificates, death certificates, records from law enforcement, social services, and pediatric and obstetric medical encounters. All cases were locally evaluated, and data was entered into NFR-CRS database. This data was then reviewed centrally. A death scene investigation was conducted for 193/211 decedents (91.5%) which included evaluation of the location and circumstances of death to aid in determining cause of death. Autopsy was performed in 202/211 decedents (95.7%). As part of the autopsy, histopathology was performed in 189/211 (89.6%) and a toxicology screen was completed in 193/211 (91.5%) of decedents. Central review of each case relied on information from the standardized case report forms and algorithm to categorize the cause of death. The SDY Case Registry made a final determination on case categorization and entered summary data into an existing web-based data collection tool, NFR-CRS [10]. The SDY Case Registry case status was considered a final adjudication. Further annotations about possible cause of death or contributory factors were based on data from NFR-CRS summaries or were obtained from an affiliated data collection system [11]. Due to Data Use Agreements (DUAs) regarding the integrity and protection of the NFR-CRS data, local and national institutional review board (IRB), and informed consent constraints, the clinical data shared with the investigators of this current study were limited. Biospecimens for DNA extraction were obtained at autopsy if consent was obtained from next-of-kin. Registry activities involving biospecimen collection and consent of surviving family members for research were approved by the IRB at the Data Coordinating Center and IRBs at participating states/jurisdictions.

### Genome sequence (GS) analysis

GS analysis was performed on DNA obtained from the SDY Case Registry using an Illumina XTen sequencer (Garvan Institute of Medical Research NSW, Australia) with a yield of > 100 GB per sample, correlating to > 30-fold coverage across the genome. The Burrows-Wheeler Aligner was used to align reads to human reference sequence GRCh37/hg19 [12]. Variants were called with either the Genome Analysis Tool Kit (GATK v3.3.0) using the MegaSeq pipeline or Sentieon joint variant calling software [13–15]. Variant call files were annotated using SnpEff [16]. Variants were annotated using ClinVar (accessed March 2022) and global and ancestry-specific allele frequencies using the Genome Aggregation Database (gnomAD) [17]. M-CAP was used to annotate variant pathogenicity [18].

### Genetic evaluation of ancestry

Ancestry was determined using principal component analysis (PCA) conducted using singular-value decomposition of ~5 million biallelic variants across the genome. The first 3 PCs were used to determine global genetic ancestry. Analyses were performed using PLINK v1.9 and R v4.1 [19].

### Gene panel analysis

Four previously established gene panels were analyzed (two epilepsy panels and two arrhythmia and cardiac function panels, Additional File 1: Tables S2 and S3): (1) the Early Infantile Epileptic Encephalopathy (EIEE)-Online Mendelian Inheritance in Man (OMIM) panel (82 genes) derived from the OMIM phenotypic series for early infantile epileptic encephalopathy, a curated epilepsy list; (2) the Epilepsy gene panel (191 genes, overlaps with the EIEE panel) is a curated list derived from the Invitae Epilepsy Panel (Invitae, San Francisco, CA); (3) CMAR1, Cardiomyopathy and Arrhythmia (gene panel including 118 arrythmia and cardiac genes previously described [7]); and (4) CMAR2, a Pan-Cardiomyopathy Arrhythmia and Cardiomyopathy Comprehensive gene panel including 143 genes with overlap with the CMAR1 panel [20]. Control genes were selected from uniformly distributed housekeeping genes [21, 22]. To create burden-matched control gene lists, a burden ratio measurement was calculated for each gene in RefSeq by counting the number of rare variants (MAF ≤ 0.005) found in the gnomAD database over the largest coding transcript of every gene divided by transcript length [17]. The mean burden ratio and standard deviation from each list were matched by randomly sampling from the whole genome (excluding genes in the test list) [21, 23].

### Artificial-intelligence (AI)-based variant prioritization

AI-based prioritization and scoring of candidate disease genes and diagnostic conditions were performed using Gene-Environment interaction analysis in Millions of samples (GEM) [24], a commercially available Fabric Enterprise platform (Fabric Genomics, Oakland, CA). Briefly, GEM aggregates data from multiple sources and clinical data sets to identify pathogenic variants relevant to phenotype terms input by the user. The GEM pipeline requires a variant call file, affection status, and human phenotype ontology (HPO) terms for each individual. A log_10_ Bayes-factor score (GEM score) was generated that calculated the extent of support for a given model using multiple lines of evidence from open-source tools and databases. A GEM score ≥ 0.69 was used to define a likely damaging genetic variant (GEM-damaging), based on the observation this threshold recovered 95% of true positive cases in a cohort of critically ill newborns undergoing rapid genome sequencing [24].

### Control cohort

The control cohort of 211 genome samples derived from the 1000 Genomes Project (https://www.internationalgenome.org/) that were variant called using the Sentieon pipeline described above against the GRCh37/hg19 human reference genome [25]. This control cohort matched the ancestry and sex composition of the SDY cohort.

### Enrichment analysis

Since detailed clinical information was lacking from the SDY cohort, we used HPO terms expected to be associated with SDY: Sudden Cardiac Death (HP:0001645), Sudden Death (HP:0001699), Cardiac arrest (HP:0001695), Cardiomyopathy (HP:0001638), Abnormal QT interval (HP:0031547), and Seizure (HP:0001250). We also performed the analysis using a control root phenotype, Phenotypic Abnormality (HP:0000118) which was used as a normalization factor to control for ontology biases and was expected to behave similarly across different cohorts.

GEM analysis was performed for each HPO term with each iteration identifying potentially causal variants for the three phenotypes described. To assess enrichment between cases and the controls, a resampling analysis was performed as previously described [23]. Briefly, for each investigated gene list (CMAR1, CMAR2, EIEE-OMIM, and Epilepsy Panels) of size *N*, 100,000 random samples of equal size were drawn from the 18,876 RefSeq genes and intersected with the corresponding GEM list of genes with a GEM score ≥ 0.69 (GEM-damaged) producing the number of GEM-damaged genes in the resampled list. Fisher exact test was used to determine if there were significant differences in the number of genes between cases and controls using the permutation test to normalize the data across runs and produce an empirical *p*-value [23]. Multiple comparison correction was performed using the false discovery rate (FDR) approach [26]. Each null distribution was scaled and centered producing *Z*-scores to directly compare the *p* values of different gene sets.

### Age at death burden analysis

Age at death dependence was correlated with the cumulative number of nonsynonymous variants in the Epilepsy or CMAR gene panels. Variants were binned by global gnomAD frequency [< 0.001, 0.001–0.01, 0.01–0.1, 0.1–0.25, 0.25–0.5]. Multivariate linear models adjusting for ancestry (PCs 1-6) using custom code based on the *lm* function in R 4.1 were regressed to test each bin. *P*-values were adjusted for multiple comparisons. An extreme value sensitivity test was performed for decedents at the ends of the rare variant distribution (gnomAD < 0.001); *p* values were adjusted for ancestry PCs 1-6.

### Variants of uncertain significance (VUS) analysis

The number of nonsynonymous, rare variants (< 0.001) found in gnomAD (V2) for each gene in the Epilepsy and CMAR1 gene panels was compiled. To determine expected number of variants in each gene for the gene set used (Epilepsy/CMAR), we created a multinomial model using the observed number of missense variants in the same gene set in gnomAD. We estimated the probability of observing a variant in each gene in the gene set as the observed number of variants in gnomAD by dividing the total number of variants in the gene set in gnomAD.

## Results

### The Sudden Death in the Young (SDY) cohort

**Fig. 1:**
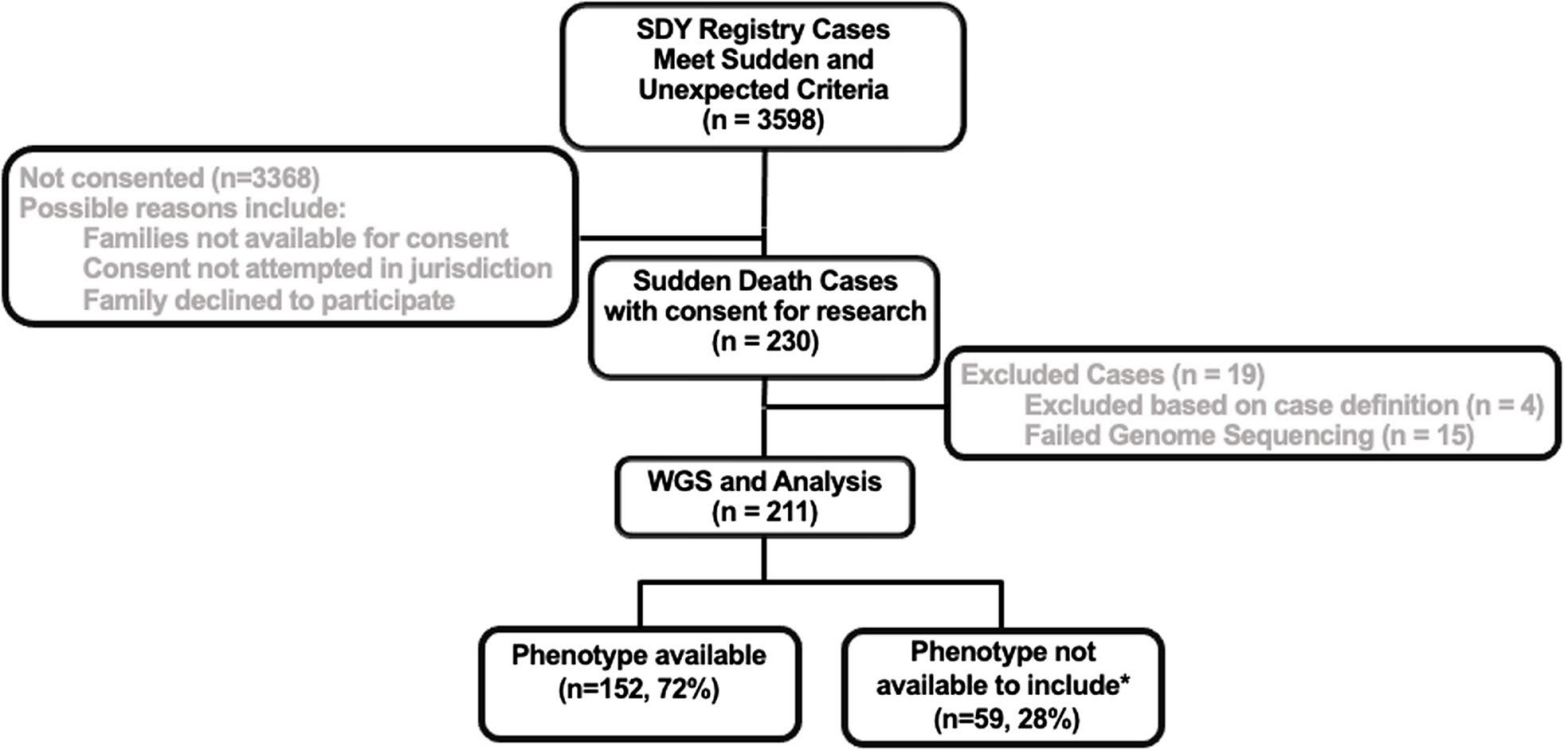
CONSORT diagram of decedents selected for genome sequencing. Phenotype data were made available by the SDY Case Registry after cause of death was determined. * Indicates the following: DUA constraints limited the sharing of detailed phenotypic data from some cases; all cases were reviewed locally and centrally by the SDY Case Registry and met inclusion criteria. These cases were considered as “Phenotype not available to include”. GS, genome sequencing

The SDY Case Registry is a joint initiative by the CDC, NIH, and the Michigan Public Health Institute. The SDY Case Registry gathers and reviews child death cases from 13 states and jurisdictions, following a standardized case reporting system, NFR-CRS. Child death review programs exist in all 50 states and provide comprehensive reviews of infant and child deaths; 13 sites were selected for SDY inclusion based on representation and willingness of local authorities to participate in the SDY Case Registry. The SDY Case Registry includes children from birth to 20 years. Inclusion requires that death be sudden, within 24 h of first symptom or death in a hospital setting after resuscitation from a cardiac arrest, and unexpected which includes subjects in good health or with an illness not reasonably expected to cause death [8]. Exclusion criteria are accidental death with only and obvious cause, homicide, suicide, overdose, and terminal illness (Additional File 1: Figure S1). From 2015 to 2019, 3598 cases met the inclusion criteria of a sudden and unexpected death and were eligible for next of kin consent. In total, 230 decedents from the SDY Case Registry were enrolled in this genetic study and underwent genome sequencing (6%) (Fig. 1). Of the 230 initially identified, 19 were excluded: 4 due to failed inclusion criteria and 15 due to failed sequencing where no additional sample was available (Fig. 1). Genomes were sequenced to 30X coverage with ~0.9 billion reads per sample [9.2 × 10^8^, 8.7x 10^8^–9.6x 10^8^] (median, IQR). Ninety-eight percent of reads were > 140 bp and all had a QC > 30.

The median age at death was < 1 year [0.33, 0.17–1.12] (median, IQR), indicating an enriched sample of infants who died in the first year of life. Genetic ancestry analysis was used to classify SDY decedents as 43% European, 29% African, 3% Asian, 16% Hispanic, and 9% with mixed ancestries. The cohort was 61% male and 39% female (Table 1). Of the 211, 152 (72%) had partial phenotype data available from the SDY Case Registry. For 59 cases (28%), individual-level clinical data, while collected and analyzed by the SDY Registry to make final adjudications, were not eligible for use by this study due to the limits of the DUAs (Table 1). All cases were locally evaluated, and data was entered into NFR-CRS database. These data were then reviewed centrally. A death scene investigation was conducted for 193/211 decedents (91.5%) which included evaluation of the location and circumstances of death to aid in determining cause of death. Autopsy was performed in 202/211 decedents (95.7%). As part of the autopsy, histopathology was performed in 189/211 (89.6%) and a toxicology screen was completed in 193/211 (91.5%) of decedents. Central review of each case relied on information from the standardized case report forms and algorithm to categorize the cause of death (Additional File 1: Table S1). The cause of death is reported here for 152 decedents, including 104 whose death remained unexplained and 48 with a known cause of death reported by the SDY Case Registry. Sudden unexplained death was the most common cited cause (43%). Detailed cause of death for the additional 59 decedents was reviewed by the central SDY Registry, but authors are not permitted to provide details here due to DUA constraints; however, these subjects all met SDY Registry inclusion criteria.

**Table 1:** Demographics of sudden death cohort (*n* = 211)

Detailed phenotype^a^	Yes	No
*n* (%)	152 (72)	59 (28)
Age at death (median [IQR])	0.33 [0.17, 1.12]	NA
Genetic ancestry (%)		
African	49 (32.2)	13 (22)
Asian	4 (2.6)	2 (3.4)
European	68 (44.7)	22 (37.3)
Hispanic	19 (12.5)	14 (23.7)
Mixed	12 (7.9)	8 (13.6)
Genetic male sex (%)	93 (61.2)	36 (61.0)

^a^All decedents are confirmed sudden death and meet SDY Case Registry inclusion criteria. Per DUA requirements, additional data were available to this study for some cases

### Pathogenic/likely pathogenic variants in cardiac and epilepsy genes

To identify variants that may have contributed to SDY, we employed gene panels to filter for rare, high impact variants. We hypothesized that the most likely candidates for SDY would be epilepsy and cardiomyopathy and arrhythmia (CMAR) gene variants. We identified variants in a 191 gene Epilepsy panel and a 118 gene CMAR panel (CMAR1) (Additional File 1: Table S2 and S3). Variants were annotated using ACMG classification and ClinVar interpretations to designate variants as pathogenic (P), likely pathogenic (LP), or variant of uncertain significance (VUS). Across these gene panels, we identified P/LP variants in ~5% of decedents (11 of 211); of these, 3/211 have a P/LP that may explain the sudden death phenotype. In the 11 individuals with a P/LP, the same *TTR* variant appeared in 4 decedents (Table 2). In the epilepsy panel, we identified 4 variants in 3 decedents, but these genes generally associate with autosomal recessive inheritance and decedents were heterozygous. Four individuals had at least one P or LP variant in genes from the CMAR1 panel, excluding the *TTR* variants. One variant was identified in the *FKRP* gene which associates with recessive disease and was heterozygous in this individual. We identified a missense *CALM3* variant designated likely pathogenic by ACMG guidelines. A pathogenic, nonsense *DSP* variant was identified that was not previously described in the literature . One individual had a frameshifting, pathogenic variant in the *KCNH2* gene, which is found in both the Epilepsy and CMAR1 panels ( Table 2).

**Table 2:** Pathogenic and likely pathogenic variants identified in epilepsy and cardiac genes

ID	Age at death (years)	Cause of death	ClinVar/criteria	GENE	gDNA	AA change	gnomAD
Epilepsy panel gene variants							
1010	0.3	Cardiac, congenital	**LP** PM2, PP5	QARS*	3:49137655 G>A	p.Arg367Cys	3.19E−05
1172	0.3	Infant suffocation	**LP** PM2,5 PP3,5	CLN8*	8:1719428 C>T	p.Arg70Cys	0
1193	20	Venous malformation	**P** PM1,2 PP2,3,5	PPT1*	1:40558081 T>G	p.Thr25Pro	0
	20	Venous malformation	**P/LP** PS4 PVS1 PM2 PP5	CSTB*	21:45194641 C>G	n.104G>C	0.0003
CMAR1 panel gene variants							
1001	0.3	Unexplained	**P** PM1,2 PP2,3,5	FKRP*	19:47259533 C>A	p.Leu276Ile	0.0014
1012	0.2	Unexplained	**P** PM1,2,5 PP2,3,5	*TTR*	18:29178618 G>A	p.Val142Ile	0.0048
1028	0.50	Unexplained	**P** PM1,2,5 PP2,3,5	*TTR*	18:29178618 G>A	p.Val142Ile	0.0048
1058	7	Unexplained	**LP** PM1,2,5 PP2,3,5	*CALM3*	19:47112212 A>G	p.Asp96Gly	0
1117	0.2	Infant suffocation	**P** PM1,2,5 PP2,3,5	*TTR*	18:29178618 G>A	p.Val142Ile	0.0048
1127	0.2	Unexplained	**P** PM1,2,5 PP2,3,5	*TTR*	18:29178618 G>A	p.Val142Ile	0.0048
1132	NA	NA	**P** PVS1 PM2 PP5	*DSP*	6:7571745 C>T	p.Gln611X	0
Epilepsy and CMAR1 panel gene variants							
1214	NA	NA	**P** PVS1 PM2,5	*KCNH2*	7:150654393 C>CAG	p.Thr371_Glu372fs	0

Variants in this table were annotated with population frequency, protein effect, literature review, and in silico modeling. Pathogenicity was determined by methods similar to ACMG standards (see the “Methods” section). *NA*, not reported in case review; *P*, pathogenic; *LP*, likely pathogenic. *Gene is generally associated with autosomal recessive inheritance and decedent is heterozygous. *AA*, amino acid

To identify P/LP in a broader set of genes without constraining to the Epilepsy and CMAR panels, we deployed GEM, an AI tool designed for whole genome-based diagnosis of Mendelian conditions [24], using HPO terms related to seizures and sudden cardiac death. Thirty-eight decedents (18%) had ClinVar-designated P/LP variants in genes linked to a Mendelian disorder (13 related to the HPO term seizure and 25 related to sudden cardiac death; Additional File 1: Table S4). Four of these decedents had compound heterozygous P/LP/VUS variants in genes associated with recessive disorders, including *CFTR* (cystic fibrosis), *BCKDHA* (maple syrup urine disease), *MPDZ* (congenital hydrocephalus-2), and *GDAP1* (Charcot-Marie-Tooth disease, type 4A).

### Damaging genetic variation in cardiac and epilepsy genes was enriched in the SDY cohort

**Fig. 2:**
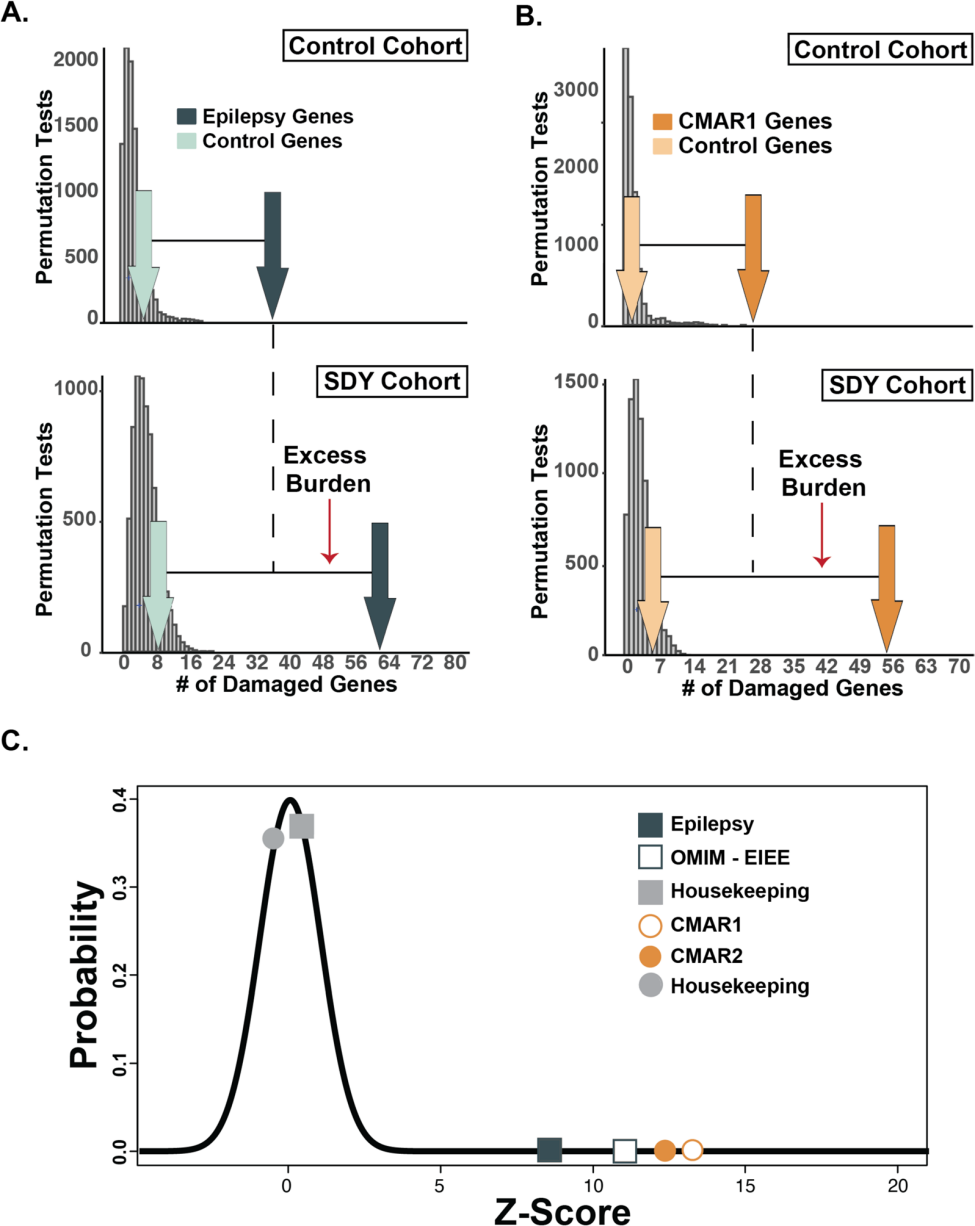
Relative enrichment of GEM-damaging variants in epilepsy and Cardiomyopathy/Arrhythmia (CMAR) genes. The SDY cohort has enriched GEM-damaging (**A**) epilepsy and (**B**) cardiac gene burden compared to a sex- and ancestry-matched control cohort. Histograms (gray bars) represent distributions of GEM-damaged genes (GEM Score ≥ 0.69) sampled randomly from RefSeq genes using a root phenotype from the SDY cohort (*n* = 211) (bottom panels) and 1000 Genomes Project Cohort (control) matched for sex and ancestry (*n* = 211) (top panels). GEM-damaged genes identified in the Epilepsy (*n* = 191 genes, green) (**A**) and CMAR1 (*n* = 118 genes, orange) (**B**) gene lists were significantly different between the SDY and control cohorts (dark arrows, epilepsy, *p* = 0.027; cardiac *p* < 0.001). Light arrows represent the number of GEM-damaged genes identified in a control housekeeping gene set. (**C**) The results in Fig. 2A and B and Additional File 1: Figure S2 were *Z*-score transformed to make the gene list findings comparable. The plot reveals a considerable enrichment in both cardiac and epilepsy gene lists (+ 8.0 to + 13.0 SDs). Black line represents the expected normal distribution

We compared the burden of potentially damaging cardiac and epilepsy genes in SDY cases versus controls, using two CMAR and two Epilepsy gene panels (see the “Methods” section). A GEM score ≥ 0.69 was used to define a likely damaging genetic variant (GEM-damaging) (see the “Methods” section). We also used a known housekeeping gene list and a random sample of genes matched for similar variant burden [22]. Figure 2 provides the distributions for the random gene samples (gray bars) and the number of genes identified for enrichment in the gene lists (dark arrows) and the burden-matched controls (light arrows). This analysis revealed an increased burden of genes with GEM-damaging variants for both Epilepsy and CMAR1 panels when compared with the control gene set (Fig. 2A and B, respectively). A similar pattern was seen when we analyzed the EIEE-OMIM and CMAR2 gene panels (Additional File 1: Figure S2). The Fisher exact-test *p*-values FDR adjusted are Epilepsy *p* = 0.027, EIEE-OMIM *p* = 0.019, CMAR1 *p* < 0.001, and CMAR2 *p* < 0.001 (Additional File 1: Table S5).

To compare the findings among the distributions shown in Fig. 2A and B, each distribution was normalized by *Z*-score transformation (Fig. 2C). The standardized *Z*-score values of the enrichment observed in the gene panels (Fig. 2C) exceeded the expected normal distribution (shown in black), indicating a strong enrichment for GEM-damaging variation in Epilepsy and CMAR genes in the SDY cohort (+ 8.0 to + 13.0 standard deviations).

### Rare variants in epilepsy genes correlated with age at death

**Fig. 3:**
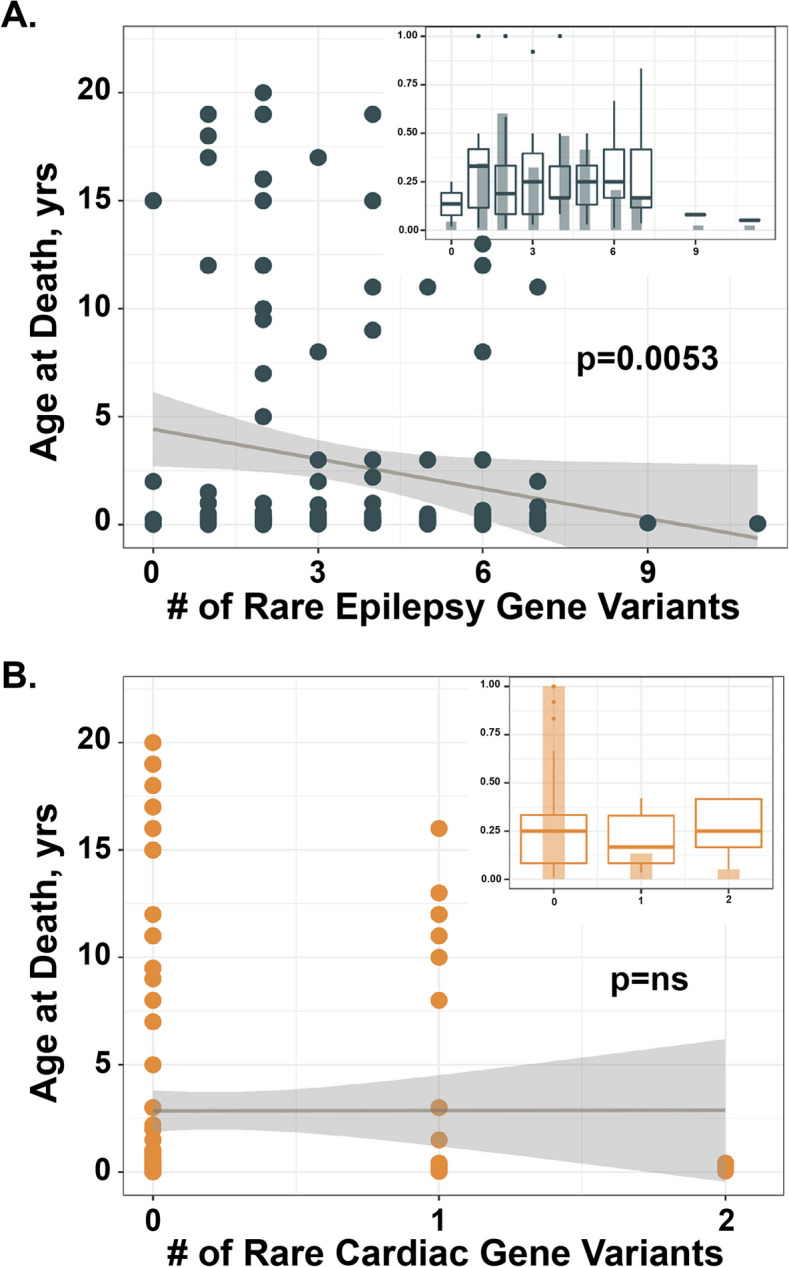
Number of rare, nonsynonymous, epilepsy gene variants correlated with younger age at death. The age at death was plotted against the number of rare variants (gnomAD allele frequency < 0.001) in either the **A** Epilepsy or **B** CMAR1 gene list. Number of variants in the epilepsy gene list significantly correlated with age at death, (*p* = 0.0053, adjusted for the first 6 principal components of ancestry). A similar analysis of rare, predicted damaging variation in the CMAR1 genes did not show association with age at death (*p* = 0.85, *p* value adjusted for ancestry). Inset is a histogram showing number of decedents < 1 year old by variant burden, box plots (black) represent decedent age distribution for each variant burden. Dark gray line = regression line of unadjusted model; dark gray shading represents 95% confidence intervals

The enrichment of GEM-predicted damaging variation in Epilepsy and CMAR genes and the few decedents with P/LP variants led us to determine if there is an association between rare Epilepsy and CMAR variants and age at death. We aggregated variants across five gnomAD allele frequency bins (< 0.001, 0.001–0.01, 0.01–0.1, 0.1–0.25, 0.25–0.5) for the Epilepsy and CMAR1 gene panels. We found that having more rare, nonsynonymous variants in Epilepsy genes was associated with younger age at death. Nonsynonymous Epilepsy variants with an allele frequency ≤ 0.001 were significantly associated with age at death (*p* = 0.0053, adjusted for ancestry) (Fig. 3A and Additional File 1: Table S6). Results remained significant (*p* = 0.026) following correction for multiple hypothesis testing for all 5 frequency bins. To confirm that these data were not driven by a few outliers, we performed a sensitivity analysis that supported the results of the main analysis (Additional File 1: Table S6). A similar analysis of rare, nonsynonymous variants in the CMAR1 gene panel did not show an association for age at death in any frequency bin (Fig. 3B). To determine if the burden of rare epilepsy (MAF ≤ 0.001) variants was driven by the SUDEP cases, we compared the burden between known SUDEP decedents (*n* = 6) and known cardiac cases (*n* = 15). We did not find any difference (*p* = 0.18, Wilcoxon rank-sum test) between these groups. It is difficult to draw conclusions with these very small sample sizes (*n* = 6, SUDEP and *n* = 15, known cardiac), however, the observation that there is a similar burden of rare epilepsy variants in decedents with SUDEP and decedents with cardiac findings suggests that these variants may be risk alleles for sudden death across this young cohort.

### Rare VUS in epilepsy and cardiac genes were enriched in the SDY cohort

**Fig. 4:**
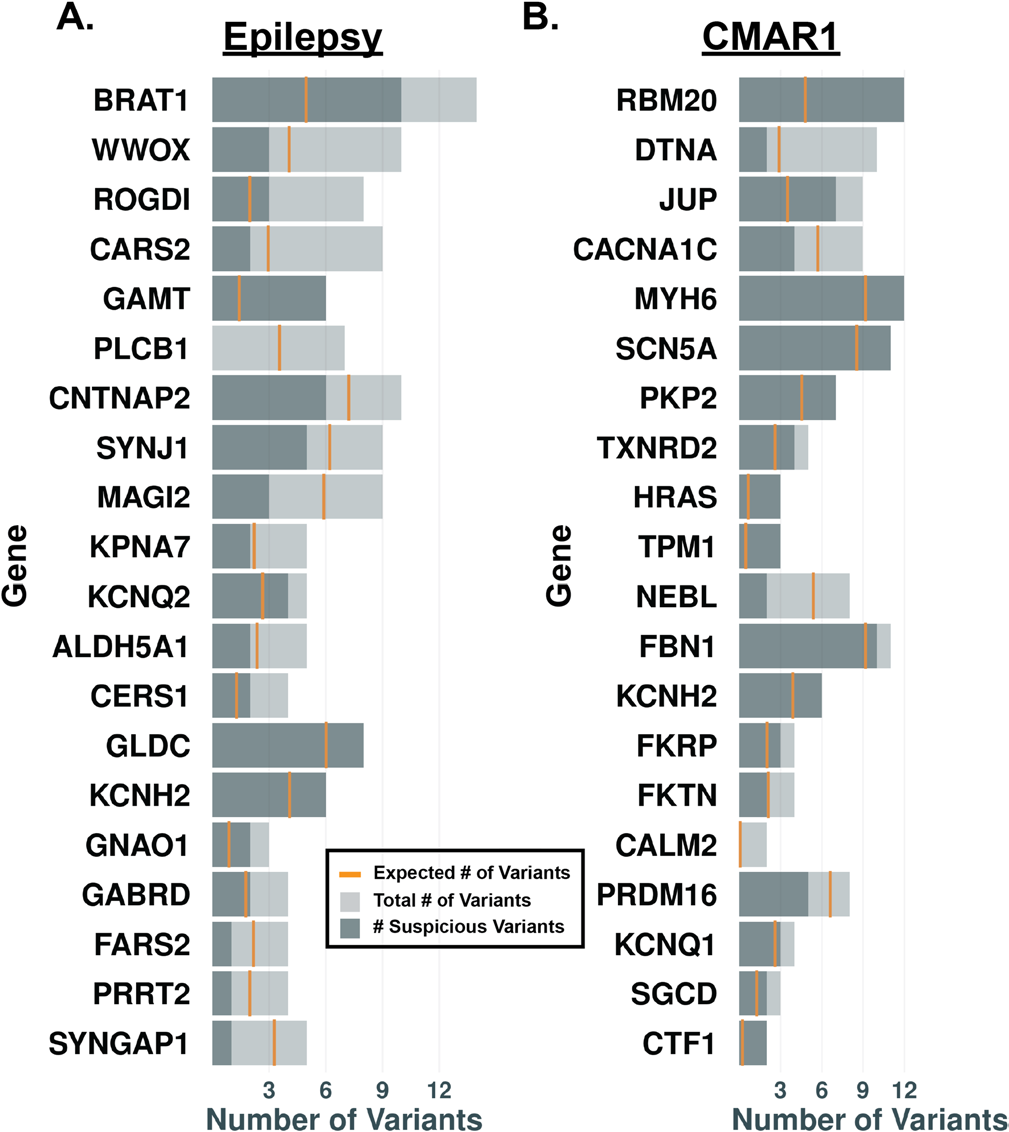
Rare VUS in Epilepsy and CMAR genes. The number of rare (< 0.001 gnomAD allele frequency) variants not classified as pathogenic or benign in ClinVar are reported for genes from either the **A** Epilepsy or **B** CMAR1 gene list (light gray bars). Variants were scored using the in silico tool M-CAP. The number of suspicious VUS for each gene (> 0.025 M-CAP score) is shown by the dark gray bars. The gold line indicates the expected number of variants based on gnomAD allele frequency data. Gene lists are ranked by the difference between the observed and expected number of variants, when ratios are approximately the same, ranking is determined by number of suspicious gene variants

We next considered if presence of a VUS might associate with SDY. VUS are rare variants with insufficient evidence to determine whether they are pathogenic or benign, and the major determinant of VUS status is rare population frequency. Figure 4 plots the number of rare, nonsynonymous variants (< 0.001 gnomAD allele frequency) not reported as pathogenic or benign in ClinVar. Variants were scored using the in silico predictor M-CAP and scored as suspicious VUS using an M-CAP score > 0.025. We plotted total variants, suspicious variants, and expected number of variants based on gnomAD distribution and age at death (3rd quartile) of each decedent, and we found that genes in both Epilepsy and CMAR1 panels had greater than expected numbers of variants. Some genes had very few suspicious variants, while others had more than expected. Those genes with greater than expected variation may potentially drive or modify risk for SDY.

## Discussion

This SDY cohort is unique for its very young age (median age < 1 year) and its diverse ancestry. We identified P/LP variants in Epilepsy and CMAR genes in ~5% of the cohort. Of the 12 variants identified in the cohort, 4 unrelated decedents carried the same *TTR* variant, Val142Ile. *TTR* encodes transthyretin, and pathogenic *TTR* variants are associated with the development of hereditary *TTR* amyloidosis. *TTR* variants are generally associated with later onset cardiomyopathy and neuropathy and increased risk of heart failure [27]. The Val142Ile variant has a global gnomAD frequency of 0.5% but a frequency of 2% in individuals of African Ancestry. In this comparatively small cohort, the *TTR* variant was present in 5% of subjects. The *TTR* Val142Ile variant confers risk for cardiac amyloidosis which typically has clinical onset after the fifth decade as heart failure and arrhythmias [28]. A role for *TTR* in early life has not been established, and larger cohort studies are needed to understand risk. Evaluating the epilepsy and CMAR panels revealed 5 P/LP variants in genes that generally associate with homozygous recessive inheritance (*QARS, CLN8, PPT1*, *CSTB*, and *FKRP*) in heterozygous decedents, indicating that these are likely incidental findings. However, these variants may contribute to the overall burden of variants that predispose to sudden death events. The remaining P/LP variants were likely causative variants and were considered actionable by the ACMG. We identified a missense *CALM3* variant designated likely pathogenic by ACMG guidelines. *CALM3* variants are associated with long QT syndrome and ventricular arrhythmias, and more recently idiopathic ventricular fibrillation and cardiomyopathy, and have been described in young sudden death cases [29]. The *CALM3* D96G variant was recently described in 2 families with LQTS from the International Calmodulinopathy Registry and is likely to have contributed to the decedent’s unexplained sudden death [30]. A pathogenic, nonsense *DSP* variant was identified that was previously undescribed in the literature. *DSP* variants have been linked to arrhythmogenic and dilated cardiomyopathies, suggesting that this pathogenic *DSP* variants may have contributed to the decedent’s sudden death, although the association of DSP truncations with young sudden death is less well established [31]. One individual had a frameshifting, pathogenic *KCNH2* variant not previously described in the literature. Pathogenic *KCNH2* variants have a strong association with long QT syndrome and a less-common association with short QT syndrome and epilepsy [32–34], and based on these prior reports, we conclude this likely had a role in sudden death.

We performed an unrestrained, genome-wide AI-driven analysis and identified 42 P/LP variants in 38 decedents (18% of decedents) suggesting that a purely gene panel-based approach may be diagnostically limited. Previous studies identified that the prevalence of P/LP gene variants is less in younger cohorts [3–5, 35], consistent with findings in our young cohort. In adults, the prevalence is likely 15–20%, whereas, in infants, our data agrees with others that the prevalence is < 10% when cardiomyopathy and arrhythmia genes are considered with a panel approach. Analysis of 278 SIDS cases for ultrarare variants in noncardiac, SIDS-susceptibility genes did not identify a monogenic basis for SIDS, even when using pathway burden analyses suggesting that there is not a strong monogenic substrate for SIDS; instead, there may be a polygenic basis for disease [36, 37].

A recent study of SDY examined decedents > 1 to < 44 years of age and specifically excluded the decedents under 1 year of age [7]. Burden analysis found more CMAR1 gene variants and an association between gene variant burden and younger age at death [7]. We examined burden in both epilepsy and cardiomyopathy and arrhythmia genes in an SDY cohort, with a median age at time of death < 1 year. We found enrichment of both epilepsy and cardiac damaging variants, but only the epilepsy gene variant burden correlated with younger age at death. This contrasts with decedents > 1 year where age at death correlates with cardiac gene variant burden [7]. There is evidence that epilepsy-related mechanisms associate with SDY pathogenesis [38, 39]. We hypothesize that rare epilepsy and cardiac variants contribute to risk susceptibility in very young victims of sudden death. Our data are in line with another study showing an increased burden in epilepsy, cardiac, and metabolic genes with an excess of rare variants in all three gene classes in a SUDP cohort [6].

### Limitations

Sudden death in the young is devasting to the surviving family. The grief and trauma surrounding SDY can adversely impact participation in research. In the present study, although 3598 decedents were eligible, only 211 decedents were available for the study. A larger more expansive cohort should enable a better assessment of the complex genetics of sudden death in the young. A larger cohort would also have allowed sub-analysis of different categories of sudden death. This study was also limited by the data use agreements and consent processes in place that prohibited the full sharing of clinical details from a subset of the decedents in the study, and thus this information could not be included in the analyses. We also did not include an analysis of copy number variants (CNV) which may have improved yield of pathogenic variation. CNV identification is challenging with short-read genome sequencing as CNVs vary in size and have a similar signature to short-read artifacts [40–43]. CNV analysis would be most accurate with long-read sequencing, which was not available in this study.

## Conclusions

Together, these findings support epilepsy etiologies for SDY, particularly in infants with a broader genetic contribution including cardiac and epilepsy variants in those  < 1 year of age. Even more relevant, the genetic associations for SDY appear to derive less from single gene P/LP variants and instead correlate with an aggregation of potentially damaging variants within an individual genome that predispose to SDY.

### Supplementary Information

**Additional File 1:** **Table S1.** Detailed Cause of Death. **Table S2.** Epilepsy Gene panels. **Table S3.** Cardiomyopathy and Arrhythmia Gene panels. **Table S4.** Pathogenic and Likely pathogenic, Mendelian variants as ranked by GEM. **Table S5.** Enrichment of variants in epilepsy or cardiac genes in the SDY cohort compared to an ancestry and sex matched 1000 genomes cohort. **Table S6.** Linear regression of age at death against number of rare epilepsy variants. **Figure S1.** Summary of exclusion and inclusion criteria for the SDY Case Registry. **Figure S2.** The SDY cohort had enriched GEM-damaging (A) epilepsy and (B) CMAR2 gene burden compared to a sex- and ancestry-matched control cohort.

## Data Availability

When permitted by IRB, DUA, and consent, phenotype and sequence data is available via dbGaP through controlled access (for privacy and legal/ethical issues) under study accession phs003221.v1.p1 (https://www.ncbi.nlm.nih.gov/projects/gap/cgi-bin/study.cgi?study_id=phs003221.v1.p1) [9]. The GEM tool is commercially available from Fabric Genomics (https://fabricgenomics.com/fabric-gem/). Additional in-house scripts will be made available upon reasonable request to the corresponding author (m.puckelwartz@northwestern.edu). Responses to such requests can be expected within 4 weeks.

## Abbreviations

SDY: Sudden death in the young
NIH: National Institutes of Health
CDC: Center for Disease Control and Prevention
NFR-CRS: National Fatality Review Case Reporting System
SIDS: Sudden infant death syndrome
SUID: Sudden unexpected infant death
SUDEP: Sudden unexplained death in epilepsy
P/LP: Pathogenic or likely pathogenic
SUDP: Sudden unexpected death in pediatrics
GS: Genome sequence
GEM: Gene-Environment interaction analysis in Millions of samples
VUS: Variant of unknown significance
gnomAD: Genome aggregation database
PCA: Principal component analysis
EIEE: Early infantile epileptic encephalopathy
OMIM: Online Mendelian inheritance in man
CMAR1: Cardiomyopathy and Arrhythmia panel 1
CMAR2: Cardiomyopathy and Arrhythmia panel 2
AI: Artificial intelligence
HPO: Human phenotype ontology
FDR: False discovery rate
