# Supplementary material for: The impact of damaging epilepsy and cardiac genetic variant burden in sudden death in the young
Source: Genome Med. 2024 Jan 16;16:13. doi: 10.1186/s13073-024-01284-w (PMC10792876; doi:10.1186/s13073-024-01284-w)
Supplement: Supplementary file 1 — Additional File 1: Table S1. Detailed Cause of Death. Table S2. Epilepsy Gene panels. Table S3. Cardiomyopathy and Arrhythmia Gene panels. Table S4. Pathogenic and Likely pathogenic, Mendelian variants as ranked by GEM. Table S5. Enrichment of variants in epilepsy or cardiac genes in the SDY cohort compared to an ancestry and sex matched 1000 genomes cohort. Table S6. Linear regression of age at death against number of rare epilepsy variants. Figure S1. Summary of exclusion and inclusion criteria for the SDY Case Registry. Figure S2. The SDY cohort had enriched GEM-damaging (A) epilepsy and (B) CMAR2 gene burden compared to a sex- and ancestry-matched control cohort. [file 13073_2024_1284_MOESM1_ESM.docx]

**SUPPLEMENT**

**Table S1. Detailed Cause of Death**

| **Cause of Death** | | **Number of Decedents (%)** | |
| --- | --- | --- | --- |
| Unexplained **(n=104, 49%)** | |  |  |
|  | Sudden unexplained death | 90 (43) |  |
|  | Possible SUDEP | 6 (3) |  |
|  | Cardiac findings of uncertain significance | 5 (2) |  |
|  | Concerning family history | 3 (1) |  |
| Explained **(n=48, 23%)** | |  |  |
|  | Infant suffocation | 15 (7) |  |
|  | Systemic viral or bacterial infection | 13 (6) |  |
|  | Asthma | 3 (1) |  |
|  | Cardiomyopathy | 3 (1) |  |
|  | Drowning | 3 (1) |  |
|  | Multisystem failure in complex medical patient | 3 (1) |  |
|  | Major vascular malformation | 2 (1) |  |
|  | Myocarditis | 2 (1) |  |
|  | Congenital heart disease | 2 (1) |  |
|  | Diabetic ketoacidosis | 1 (<1) |  |
|  | Pulmonary embolism | 1 (<1) |  |
| *SDY Registry Review Completed | | 59 (28) |  |
| **TOTAL** | | **211** |  |

SUDEP = Sudden Unexplained Death in Epilepsy. Cardiac findings of uncertain significance included findings of cardiomyopathy without sufficient criteria for diagnosis (2 decedents), possible coronary artery disease (2), and aortic valve abnormality with left ventricular hypertrophy (1). Concerning family history included long QT syndrome, sudden unexplained death in a sibling, and family history of heart attack and stroke < 50 years of age, each in 1 decedent. Congenital heart disease included hypoplastic left heart syndrome (1) and anomalous left coronary artery (1). *Per DUAs, detailed cause of death is not eligible to be listed here, decedents met inclusion requirements for sudden death.

**Table S2. Epilepsy Gene panels**

| **Gene** | **Epilepsy n=191** | **OMIM EIEE n=82** |
| --- | --- | --- |
| *AARS1* |  | X |
| *ABAT* | X |  |
| *ACTL6B* |  | X |
| *ADAM22* |  | X |
| *ADSL* | X |  |
| *ALDH5A1* | X |  |
| *ALDH7A1* | X |  |
| *ALG13* | X | X |
| *AMT* | X |  |
| *AP3B2* |  | X |
| *ARHGEF9* | X | X |
| *ARHGEF15* | X | X |
| *ARV1* |  | X |
| *ARX* | X | X |
| *ATP1A2* | X |  |
| *ATP1A3* | X |  |
| *ATP6AP2* | X |  |
| *ATRX* | X |  |
| *BRAT1* | X |  |
| *C12orf57* | X |  |
| *CACNA1A* | X | X |
| *CACNA1E* |  | X |
| *CACNA1H* | X |  |
| *CACNA2D2* | X |  |
| *CACNB4* | X |  |
| *CAD* |  | X |
| *CARDS2* | X |  |
| *CASK* | X |  |
| *CASR* | X |  |
| *CDKL5* | X | X |
| *CERS1* | X |  |
| *CHD2* | X |  |
| *CHRNA2* | X |  |
| *CHRNA4* | X |  |
| *CHRNA7* | X |  |
| *CHRNB2* | X |  |
| *CLCN4* | X |  |
| *CLN2* | X |  |
| *CLN3* | X |  |
| *CLN5* | X |  |
| *CLN6* | X |  |
| *CLN8* | X |  |
| *CNTN2* | X |  |
| *CNPY3* |  | X |
| *CNTNAP2* | X |  |
| *CPA6* | X |  |
| *CPLX1* |  | X |
| *CSTB* | X |  |
| *CTSD* | X |  |
| *CUX2* |  | X |
| *CYFIP2* |  | X |
| *DENND5A* |  | X |
| *DEPDC5* | X |  |
| *DIAPH1* | X |  |
| *DNAJC5* | X |  |
| *DNM1* | X | X |
| *DOCK7* | X | X |
| *DYRK1A* | X |  |
| *EEF1A2* | X | X |
| *EFHC1* | X |  |
| *EHMT1* | X |  |
| *EPM2A* | X |  |
| *FARS2* | X |  |
| *FASN* | X |  |
| *FGF12* |  | X |
| *FLNA* | X |  |
| *FOLR1* | X |  |
| *FOXG1* | X |  |
| *FRRS1L* | X | X |
| *GABBR2* | X | X |
| *GABRA1* | X | X |
| *GABRA2* | X | X |
| *GABRB3* | X | X |
| *GABRA5* |  | X |
| *GABRB1* |  | X |
| *GABRB3* |  | X |
| *GABRD* | X |  |
| *GABRG2* | X | X |
| *GAL* | X |  |
| *GAMT* | X |  |
| *GATM* | X |  |
| *GCSH* | X |  |
| *GLDC* | X |  |
| *GLRA1* | X |  |
| *GLS* |  | X |
| *GNAO1* | X | X |
| *GOSR2* | X |  |
| *GPHN* | X |  |
| *GRIN1* | X |  |
| *GRIN2A* | X |  |
| *GRIN2B* | X | X |
| *GRIN2D* |  | X |
| *GUF1* |  | X |
| *HCN1* | X | X |
| *HNRNPU* | X | X |
| *IER3IP1* | X |  |
| *IQSEC2* | X |  |
| *ITPA* | X | X |
| *JMJD1C* | X |  |
| *KANSL1* | X |  |
| *KCNA1* | X |  |
| *KCNA2* | X | X |
| *KCNB1* | X | X |
| *KCNC1* | X |  |
| *KCND2* | X |  |
| *KCNH2* | X |  |
| *KCNH5* | X |  |
| *KCNJ10* | X |  |
| *KCNMA1* | X |  |
| *KCNQ2* | X | X |
| *KCNQ3* | X |  |
| *KCNT1* | X |  |
| *KCNT2* |  | X |
| *KCTD7* | X |  |
| *KPNA7* | X |  |
| *LGI1* | X |  |
| *LIAS* | X |  |
| *LMNB2* | X |  |
| *MAGI2* | X |  |
| *MBD5* | X |  |
| *MDH2* |  | X |
| *MECP2* | X |  |
| *MEF2C* | X |  |
| *MFSD8* | X |  |
| *MTOR* | X |  |
| *NECAP1* | X | X |
| *NEDD4L* | X |  |
| *NEUROD2* |  | X |
| *NEXMIF* | X |  |
| *NGLY1* | X |  |
| *NHLRC1* | X |  |
| *NPRL3* | X |  |
| *NR2F1* | X |  |
| *NRXN1* | X |  |
| *NTRK2* |  | X |
| *PACS1* | X |  |
| *PACS2* |  | X |
| *PARS2* |  | X |
| *PCDH19* | X | X |
| *PHACTR1* |  | X |
| *PIGA* | X | X |
| *PIGB* |  | X |
| *PIGG* | X |  |
| *PIGN* | X |  |
| *PIGO* | X |  |
| *PIGP* |  | X |
| *PIGQ* | X | X |
| *PIGV* | X |  |
| *PIK3AP1* | X |  |
| *PLCB1* | X | X |
| *PNKD* | X |  |
| *PNKP* | X | X |
| *PNPO* | X |  |
| *POLG* | X |  |
| *PPT1* | X |  |
| *PRDM8* | X |  |
| *PRICKLE1* | X |  |
| *PRICKLE2* | X |  |
| *PRIMA1* | X |  |
| *PRRT2* | X |  |
| *PTEN* | X |  |
| *PURA* | X |  |
| *QARS* | X |  |
| *RANBP2* | X |  |
| *RBFOX1* | X |  |
| *RBFOX3* | X |  |
| *RELN* | X |  |
| *RHOBTB2* |  | X |
| *RNF13* |  | X |
| *ROGD1* | X |  |
| *RYR3* | X |  |
| *SATB2* | X |  |
| *SCARB2* | X |  |
| *SCN1A* | X | X |
| *SCN1B* | X | X |
| *SCN2A* | X | X |
| *SCN3A* | X | X |
| *SCN8A* | X | X |
| *SCN9A* | X | X |
| *SERPINI1* | X |  |
| *SETD2* | X |  |
| *SGCE* | X |  |
| *SIK1* | X | X |
| *SCL12A5* | X | X |
| *SLC13A5* | X | X |
| *SLC19A3* | X |  |
| *SLC1A2* |  | X |
| *SLC25A12* | X | X |
| *SLC25A22* | X | X |
| *SLC2A1* | X |  |
| *SLC35A2* | X | X |
| *SLC35A3* | X |  |
| *SLC6A1* | X |  |
| *SLC6A8* | X |  |
| *SLC9A6* | X |  |
| *SMC1A* | X |  |
| *SNAP25* | X |  |
| *SNX27* | X |  |
| *SPATA5* | X |  |
| *SPTAN1* | X | X |
| *SRPX2* | X |  |
| *ST3GAL3* | X | X |
| *ST3GAL5* | X |  |
| *STRADA* | X |  |
| *STX1B* | X |  |
| *STXBP1* | X | X |
| *SYN1* | X |  |
| *SYNGAP1* | X | X |
| *SYNJ1* | X | X |
| *SZT2* | X | X |
| *TBC1D24* | X | X |
| *TBL1XR1* | X |  |
| *TCF4* | X | X |
| *TPK1* | X |  |
| *TPP1* | X |  |
| *TRAK1* |  | X |
| *TSC1* | X |  |
| *TSC2* | X |  |
| *UBA5* |  | X |
| *UBE3A* | X |  |
| *WDR45* | X |  |
| *WWOX* | X | X |
| *YWHAG* |  | X |
| *ZDHHC9* | X |  |
| *ZEB2* | X |  |

**Table S3. Cardiomyopathy and Arrhythmia Gene panels**

| **Gene** | **CMAR1 n=118** | **CMAR2 n=143** |
| --- | --- | --- |
| *A2ML1* | *X* |  |
| *ABCC9* | *X* | *X* |
| *ACTC1* | *X* | *X* |
| *ACTN2* | *X* | *X* |
| *AGL* |  | *X* |
| *AKAP9* | *X* | *X* |
| *ALMS1* | *X* |  |
| *ANK2* | *X* | *X* |
| *ANKRD1* | *X* | *X* |
| *BAG3* | *X* | *X* |
| *BRAF* | *X* | *X* |
| *CACNA1C* | *X* | *X* |
| *CACNA2D1* | *X* | *X* |
| *CACNB2* | *X* | *X* |
| *CALM1* | *X* | *X* |
| *CALM2* | *X* | *X* |
| *CALM3* | *X* | *X* |
| *CALR3* |  | *X* |
| *CASQ2* | *X* | *X* |
| *CAV3* | *X* | *X* |
| *CBL* | *X* |  |
| *CHRM3* | *X* | *X* |
| *CRYAB* | *X* | *X* |
| *CSRP3* | *X* | *X* |
| *CTF1* | *X* | *X* |
| *CTNNA3* | *X* | *X* |
| *DEPDC5* |  | *X* |
| *DES* | *X* | *X* |
| *DMD* | *X* | *X* |
| *DOLK* | *X* | *X* |
| *DSC2* | *X* | *X* |
| *DSG2* | *X* | *X* |
| *DSP* | *X* | *X* |
| *DTNA* | *X* | *X* |
| *EMD* | *X* | *X* |
| *EYA4* | *X* | *X* |
| *FBN1* | *X* |  |
| *FHL1* | *X* | *X* |
| *FHL2* | *X* | *X* |
| *FKRP* | *X* | *X* |
| *FKTN* | *X* | *X* |
| *FLNC* | *X* | *X* |
| *GAA* |  | *X* |
| *GATA4* | *X* | *X* |
| *GATA6* | *X* |  |
| *GATAD1* | *X* | *X* |
| *GJA5* |  | *X* |
| *GLA* | *X* | *X* |
| *GPD1L* | *X* | *X* |
| *HCN4* | *X* | *X* |
| *HRAS* | *X* | *X* |
| *ILK* | *X* | *X* |
| *JPH2* | *X* | *X* |
| *JUP* | *X* | *X* |
| *KCNA5* |  | *X* |
| *KCND3* | *X* | *X* |
| *KCNE1* | *X* | *X* |
| *KCNE2* | *X* | *X* |
| *KCNE3* | *X* | *X* |
| *KCNE5* | *X* | *X* |
| *KCNH2* | *X* | *X* |
| *KCNJ2* | *X* | *X* |
| *KCNJ5* | *X* | *X* |
| *KCNJ8* | *X* | *X* |
| *KCNK3* |  | *X* |
| *KCNQ1* | *X* | *X* |
| *KCNQ2* |  | *X* |
| *KCNQ3* |  | *X* |
| *KCNT1* |  | *X* |
| *KRAS* | *X* | *X* |
| *LAMA4* | *X* | *X* |
| *LAMP2* | *X* | *X* |
| *LDB3* | *X* | *X* |
| *LMNA* | *X* | *X* |
| *LRRC10* |  | *X* |
| *MAP2K1* | *X* | *X* |
| *MAP2K2* | *X* | *X* |
| *MOG1* | *X* |  |
| *MTND1* |  | *X* |
| *MTND5* |  | *X* |
| *MTND6* |  | *X* |
| *MTTD* |  | *X* |
| *MTTG* |  | *X* |
| *MTTH* |  | *X* |
| *MTTI* |  | *X* |
| *MTTK* |  | *X* |
| *MTTL1* |  | *X* |
| *MTTL2* |  | *X* |
| *MTTM* |  | *X* |
| *MTTQ* |  | *X* |
| *MTTS1* |  | *X* |
| *MTTS2* |  | *X* |
| *MYBPC3* | *X* | *X* |
| *MYH6* | *X* | *X* |
| *MYH7* | *X* | *X* |
| *MYL2* | *X* | *X* |
| *MYL3* | *X* | *X* |
| *MYL4* | *X* | *X* |
| *MYLK2* | *X* | *X* |
| *MYOM1* | *X* | *X* |
| *MYOZ2* | *X* | *X* |
| *MYPN* | *X* | *X* |
| *NEBL* | *X* | *X* |
| *NEXN* | *X* | *X* |
| *NKX2-5* | *X* | *X* |
| *NPPA* |  | *X* |
| *NRAS* | *X* | *X* |
| *PCDH19* |  | *X* |
| *PDLIM3* | *X* | *X* |
| *PKP2* | *X* | *X* |
| *PLEKHM2* |  | *X* |
| *PLN* | *X* | *X* |
| *PRDM16* | *X* | *X* |
| *PRKAG2* | *X* | *X* |
| *PRRT2* |  | *X* |
| *PTPN11* | *X* | *X* |
| *RAF1* | *X* | *X* |
| *RANGRF* |  | *X* |
| *RASA1* | *X* |  |
| *RBM20* | *X* | *X* |
| *RIT1* | *X* |  |
| *RYR2* | *X* | *X* |
| *SCN10A* | *X* | *X* |
| *SCN1A* |  | *X* |
| *SCN1B* | *X* | *X* |
| *SCN2B* | *X* |  |
| *SCN3B* | *X* | *X* |
| *SCN4B* | *X* | *X* |
| *SCN5A* | *X* | *X* |
| *SCN8A* |  | *X* |
| *SCN9A* |  | *X* |
| *SDHA* | *X* |  |
| *SGCD* | *X* | *X* |
| *SHOC2* | *X* |  |
| *SLC22A5* |  | *X* |
| *SLC2A1* |  | *X* |
| *SLMAP* |  | *X* |
| *SNTA1* | *X* | *X* |
| *SOS1* | *X* | *X* |
| *SPRED1* | *X* |  |
| *TAZ* | *X* | *X* |
| *TCAP* | *X* | *X* |
| *TGFB3* | *X* | *X* |
| *TMEM43* | *X* | *X* |
| *TMPO* | *X* | *X* |
| *TNNC1* | *X* | *X* |
| *TNNI3* | *X* | *X* |
| *TNNT2* | *X* | *X* |
| *TPM1* | *X* | *X* |
| *TRDN* | *X* | *X* |
| *TRPM4* |  | *X* |
| *TTN* | *X* | *X* |
| *TTR* | *X* | *X* |
| *TXNRD2* | *X* | *X* |
| *VCL* | *X* | *X* |

**Table S4. Pathogenic and Likely pathogenic, Mendelian variants as ranked by GEM**

| ID | Age (yrs) | Cause of Death | CV | GENE | gDNA | AA Change | gAD  Freq | | MIM |
| --- | --- | --- | --- | --- | --- | --- | --- | --- | --- |
| HPO Term: Seizure | | | | | | | | | |
| 1098 | 9 | SUDEP | **P**  PVS1  PM2  PP5 | *ANKRD11* | 16:89345623 G>A | Gln2443Ter | 0 | | 148050 |
| 1079 | 0.3 | UnE | **P**  PVS1  PM2  PP5 | *BCKDHA* | 19:41916543 AC>A | Arg40GlyfsTer23 | 6.5E-05 | | 248600 |
|  |  |  | **VUS**  PM2  PP2 | *BCKDHA* | 19:41916645 A>G | Asn71Ser | 6.4E-05 | | 248600 |
| 1199 | 1 | E | **P**  PS1  PM4  PM2  PP5 | *CFTR* | 7:117199645 TCTdel | Phe508del | 0.0019 | | 219700 |
|  |  |  | **P**  PM1  PP2  PM5  PP3  PP5 | *CFTR* | 7:117188877 G>T | Lys464Asn | 0.0072 | 219700 | |
| 1048 | 10 | E | **P**  PM1  PP2  PM2  PP3  PP5 | *DHDDS* | 1:26764719 A>G | Lys42Glu | 0 | 617836 | |
| 1172 | 0.25 | I.S> | **P**  PP3  PM2  PM5  PP2  PP5 | *GJB2* | 13:20763626 C>T | Arg32His | 0 | 148210 | |
| 1189 | NA | NA | **P**  PM2  PM4  PP5 | *KISS1R* | 19:920746 T>A | Ter399Argext*? | 0 | 176400 | |
| 1139 | 1 | E | **P**  PVS1  PM2  PP5 | *MC4R* | 18:58038600 A>T | Leu328Ter | 0 | 618406 | |
| 1152 | 0.03 | NA | P  PVS1  PM2  PP5 | *MPDZ* | 9:13126766 CT>C | Gln1490Arg  fsTer19 | 0.0007 | 615219 | |
|  |  |  | **VUS**  PM2  BP4 | *MPDZ* | 9:13175799 C>T | Glu1003Lys | 0.0006 | 615219 | |
| 1190 | 0.3 | E | **LP**  PM2  BP6 | *MYOCD* | 17:12655857 A>G | Ile418Val | 0.0026 | 618719 | |
| 1069 | 0.17 | I.S. | **P**  PVS1  PM2  PP5 | *NEK1* | 4:170482671 C>T | Trp409Ter | 6.5E-05 | 617892 | |
| 1035 | 0.08 | UnE | **P**  PM2  BS2 | *SRPX2* | X:99917224 A>C | Tyr72Ser | 0 | 300643 | |
| 1023 | 12 | UnE | **P**  PVS1  PM2 | *TOR1A* | 9:132576340 TCTCdel | Glu303Ter | 0 | 128100 | |
| 1107 | NA | NA | **LP**  PM2  PP2  PP5 | *TREX1* | 3:48508721 G>A | Ala223Thr | 0 | 192315 | |
| HPO Term: SDY | | | | | | | | | |
| 1209 | NA | NA | **P**  PVS1  PM2  PP5 | *ANO5* | 11:22242647--A | Asn64Lys  fsTer15 | 0 | | 166260 |
| 1019 | NA | NA | **P**  PVS1  PM2  PP5 | *ANO5* | 11:22257823 G>A | splice donor | 6.5E-05 | | 166260 |
| 1166 | NA | NA | **P**  PVS1  PM2  PP5 | *BARD1* | 2:215595215 G>A | Arg641Ter | 0 | 114480 | |
| 1180 | NA | NA | **P**  PVS1  PM2  PP5 | *BARD1* | 2:215645402 A>C | Leu399Ter | 0 | 114480 | |
| 1058 | 7 | UnE | **LP**  PM2  PM5  PM1  PP2  PP3  PP5 | *CALM3* | 19:47112212 A>G | Asp132Gly | 0 | 618782 | |
| 1203 | NA | NA | **P**  PVS1  PM2  PP5 | *CAPN3* | 15:42695962 TG>T | Asp591IlefsTer4 | 0 | 618129 | |
| 1059 | NA | NA | **P**  PM2  PP3  PP2  PP5 | *CAPN3* | 15:42703497 C>A | Ala798Glu | 0 | 618129 | |
| 1161 | 0.03 | UnE | **P**  PM1  PP2  PM2  PM5  PP3  PP5 | *CAPN3* | 15:42693950 G>A | Arg489Gln | 0.0003 | 618129 | |
| 1132 | NA | NA | **P**  PVS1  PM2  PP5 | *DSP* | 6:7571745 C>T | Gln611Ter | 0 | 607450 | |
| 1085 | NA | NA | **LP**  PM2  PP3  PP2  PP5 | *F11* | 4:187201717 T>C | Leu373Ser | 0 | 612416 | |
| 1119 | NA | NA | **P**  PVS1  PM2  PP5 | *FGA* | 4:155508672 G>A | Arg168Ter | 0 | 105200 | |
| 1050 | NA | NA | **P**  PP3  PM2  PP2  PP5 | *G6PD* | X:153761337 C>T | Val321Met | 0 | 300908 | |
| 1026 | 0.3 | UnE | **P**  PVS1  PM2  PP5 | *GDAP1* | 8:75275175 C>G | Ser194Ter | 0 | 214400 | |
|  |  |  | **VUS**  PM2  PM1  PP2  BP4 | *GDAP1* | 8:75263650 A>G | Ile87Val | 0 | 214400 | |
| 1140 | 0.3 | I.S. | **LP**  PP1  PS3  PS4  PP3  PM2  PP2  PP5 | *ITGB3* | 17:45362012 C>T | Pro189Ser | 0 | 187800 | |
| 1214 | NA | NA | **P**  PVS1  PM2  PP5 | *KCNH2* | 7:150654393 C>CAG | Glu372Leu  fsTer63 | 0 | 613688 | |
| 1077 | 0.08 | UnE | **LP**  PM1  PP2  PM2  PP3 | *LDLR* | 19:11230828 G>A | Gly636Ser | 0 | 143890 | |
| 1133 | NA | NA | **P**  PP1  PS4  PS3  PM2  PM5  PP3  PP2  PP5 | *LDLR* | 19:11218160 G>A | Asp304Asn | 0 | 143890 | |
| 1018 | 0.42 | UnE | **P**  PM1  PP2  PM2  PM5  PP5 | *NALCN* | 13:101944423 A>G | Ile322Thr | 0 | 616266 | |
| 1034 | NA | NA | **P**  PVS1  PM2  PP5 | *RYR1* | 19:38983277 G>A | splice donor | 0 | 255310 | |
| 1107 | NA | NA | **LP**  PVS1  PM2  PP5 | *SCN4A* | 17:62026868 A>T | Tyr958Ter | 0 | 168300 | |
| 1065 | 0.42 | NA | **P**  PVS1  PM2  PP5 | *SPG7* | 16:89598370 G>GC | Gly352Arg  fsTer44 | 0.0003 | 607259 | |
| 1028 | 0.5 | UnE | **P**  PM1  PP2  PM2  PM5  PP3  PP5 | *TTR* | 18:29178618 G>A | Val142Ile | 0.0172 | 105210 | |
| 1117 | 0.17 | I.S. | **P**  PM1  PP2  PM2  PM5  PP3  PP5 | *TTR* | 18:29178618 G>A | Val142Ile | 0.0172 | 105210 | |
| 1038 | 0.25 | I.S> | **P**  PVS1  PM2  PP5 | *VIM* | 10:17271434 TC>T | Val6CysfsTer26 | 0 | 116300 | |
| 1010 | 0.25 | E, cardiac | **LP**  PM2  PP2  BP4  PP5 | *VWF* | 12:6080863 C>T | Val2484Ile | 6.5E-05 | 193400 | |

NA= Not reported in case review. I.S. = infant suffocation; UnE = unexplained; E= explained; CV=ClinVar; AA = amino acid; gAD = gnomAD.

**Table S5. Enrichment of variants in epilepsy or cardiac genes in the SDY cohort compared to an ancestry and sex matched 1000 genomes cohort.**

| GENE LIST | PHENOTYPE TERMS | |  | |
| --- | --- | --- | --- | --- |
|  | **Seizure** | |  | |
|  | **SDY** | **1000 Genomes Project** | **Genes** | **Adjusted p-Value*** |
| Epilepsy | 62 | 36 | 191 | **0.027** |
| EIEE-OMIM | 38 | 16 | 82 | **0.019** |
|  | **Cardiac** | |  |  |
| CMAR1 | 55 | 27 | 118 | **<0.001** |
| CMAR2 | 58 | 31 | 143 | **<0.001** |

***P values adjusted for multiple comparisons using the FDR adjustment.**

**Table S6. Linear regression of age at death against number of rare epilepsy variants*.**

| Model | Coefficient | P value |
| --- | --- | --- |
| Adjusted (Ancestry PCs 1-6) | -0.647 | 0.0053 |
| Unadjusted | -0.458 | 0.0387 |
| Adjusted log | -0.159 | 0.0494 |
|  |  |  |
| *Extreme value sensitivity analysis* |  |  |
| Removal of cases with more than 9 variants (Adjusted PCs) | -0.636 | 0.0113 |
| Removal of cases with more than 9 variants and less than 1 (Adjusted PCs) | -0.638 | 0.0137 |
| Removal of cases with 0 rare epilepsy(<0.001) variants (Adjusted PCs) | -0.650 | 0.0063 |

*Rare epilepsy variants = nonsynonymous variants identified in the Epilepsy gene panel with an allele frequency <0.001 in gnomAD.

**
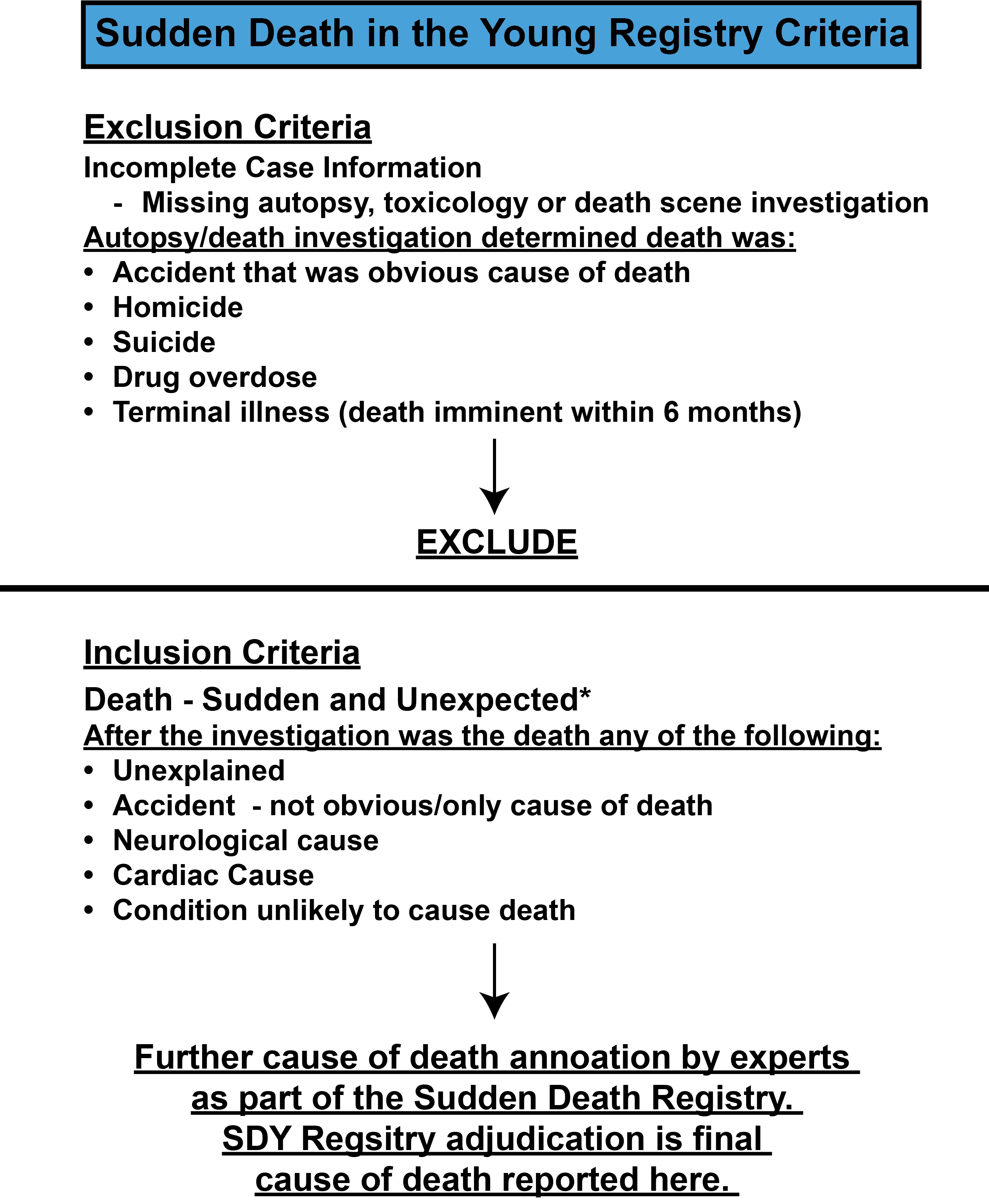
**

**Figure S1. Summary of exclusion and inclusion criteria for the SDY Case Registry.** The SDY Case Registry uses an algorithm to classify cases which relies on extensive information about the death (8). Basic inclusion/exclusion criteria are shown here. After inclusion, deaths are further categorized by a panel of experts assembled by the Sudden Death in the Young Case Registry (8). Categorized cause of death is considered a final adjudication and is reported here in Table S1. *Sudden and unexpected = within 24 hours of first symptom or death in a hospital setting after resuscitation from a cardiac arrest and unexpected which includes individuals in good health or with an illness not reasonably expected to cause death.


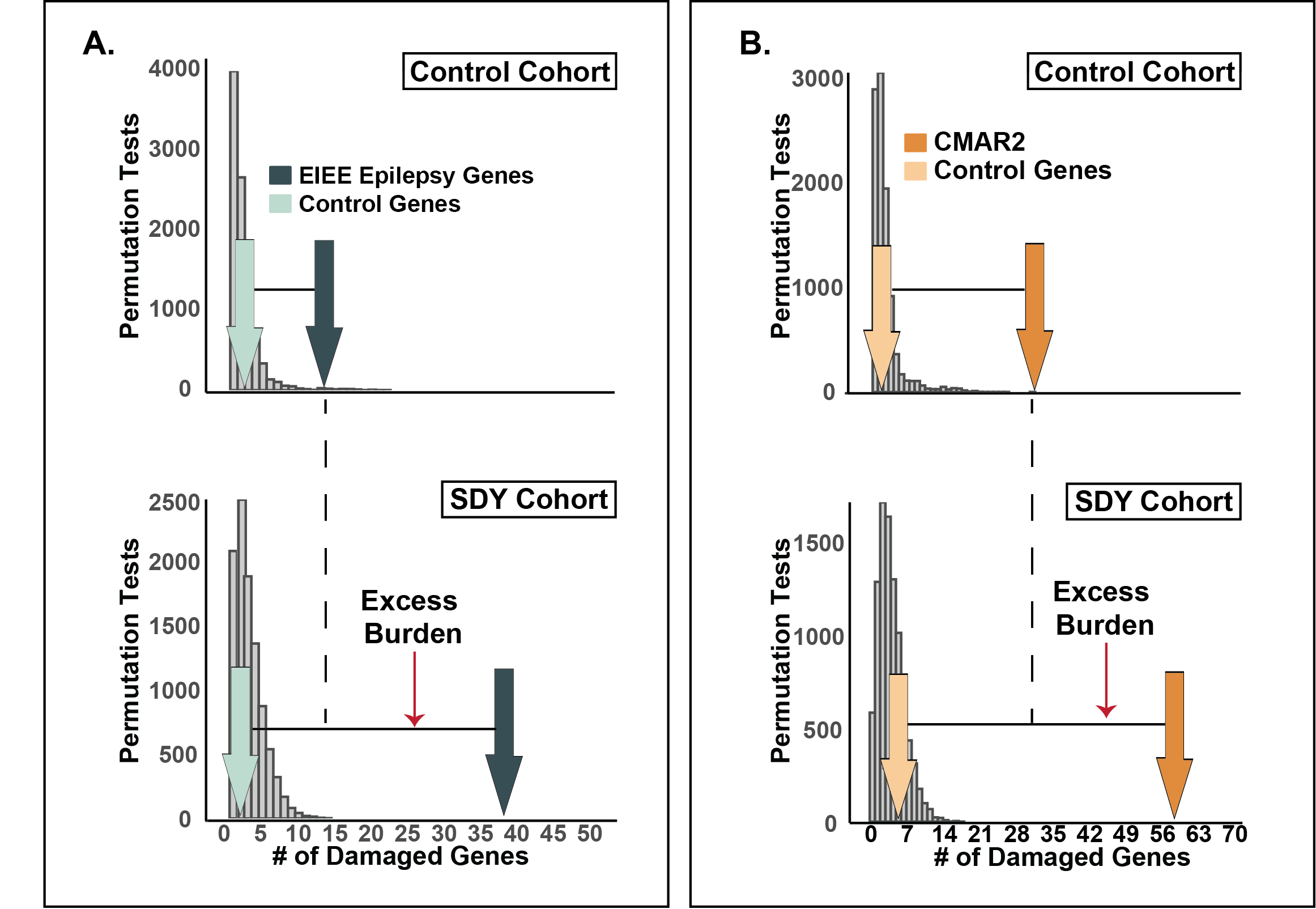


**Figure S2. The SDY cohort had enriched GEM-damaging (A) epilepsy and (B) CMAR2 gene burden compared to a sex- and ancestry-matched control cohort**. Histograms (gray bars) represent distributions of GEM-damaged genes (GEM Score >0.69) sampled randomly from RefSeq genes using a root phenotype from the SDY cohort (n=211) (bottom panels) and 1000 Genomes Project Cohort (control) matched for sex and ancestry (n=211) (top panels). GEM-damaged genes identified in the Epilepsy EIEE (n=82 genes, green) **(A)** and CMAR2 (n=143 genes, orange) **(B)** gene lists were significantly different between the SDY and control cohorts (dark arrows, epilepsy, p=0.019; cardiac p<0.001). Light arrows represent the number of GEM-damaged genes identified in a control gene set.
